# Xenoperfusion—The Transformative Role of Machine Perfusion in Xenotransplantation

**DOI:** 10.1111/xen.70131

**Published:** 2026-05-02

**Authors:** Florian Huwyler, Simon Stoerzer, Pierre‐Alain Clavien, Mark W. Tibbitt

**Affiliations:** ^1^ Macromolecular Engineering Laboratory ETH Zurich Zurich Switzerland; ^2^ Hub For Translational Research and Liver/GI Health University of Zurich, ETH Zurich, University of Fribourg Zurich Switzerland; ^3^ Department of Surgery and Transplantation University of Zurich Zurich Switzerland

## Abstract

Recent advances in xenotransplantation have gained substantial public and clinical attention as genetically modified porcine organs are now being transplanted into living human recipients. While only case reports have been published to date, the first clinical trials for kidney xenotransplantation are now ongoing. This transition to clinical practice presents multiple implementation challenges for establishing scalable transplant programs while ensuring patient safety. Machine perfusion is expected to play a critical role in addressing these challenges by serving as a central platform for organ preservation, assessment, transport, and therapeutic intervention. Given the limited number of designated pathogen‐free (DPF) breeding facilities, regional and international organ transport depends on robust preservation strategies during transit. Additionally, perfusion devices enable essential pre‐transplant screening for zoonotic pathogens, a crucial safety measure unique to xenotransplantation. Further, given recent developments that allow for multi‐day perfusion of grafts, wild‐type grafts could potentially be genetically modified while being perfused *ex situ*. Beyond these perfusion modalities of isolated whole organs, machine perfusion offers a new therapeutic approach for patients with acute liver failure. Here, cross‐circulation between a perfused genetically modified porcine organ and the patient can provide temporary liver replacement therapy. This mini‐review summarizes the transformative potential of machine perfusion technology in clinical xenotransplantation with a focus on livers.

## 1 Introduction

End‐stage organ diseases are still associated with high mortality and morbidity due to a global shortage of transplantable grafts [1, 2]. The increasing demand for solid organ transplantation aggravates the situation and is primarily driven by rising end‐stage organ disease prevalence due to lifestyle changes, population aging, and expanded transplant indications [3, 4, 5]. At the same time, increasing disease prevalence leads to a reduction of transplantable grafts—more grafts are injured or diseased upon organ donation and cannot be transplanted safely. This leads to high discard rates of 80% for lungs, 70% for hearts, and 40% for livers in the US, while similar trends can be observed globally [6]. Therefore, many patients remain on waiting lists where they eventually die, or the disease has progressed to the point that patients are removed from the waiting list as they are too sick to be transplanted.

To alleviate this devastating situation for patients, there have been mainly three approaches to offer better treatments to patients. First, political and societal changes have been implemented to spread awareness, increase willingness for living and deceased donation, or even change donation modality to *opt‐out*, where anyone is a donor by default [7]. While these measures increase the amount of organ donors, many of the donated grafts will still not be transplantable, motivating the development of additional clinically relevant strategies to increase the number of transplantable grafts.

Clinical and research efforts have focused on safely increasing organ utilization in the past decade. This was mainly enabled by broader implementation of dynamic organ preservation via machine perfusion systems. These are typically applied either in situ, for normothermic regional perfusion (NRP) [8], or *ex situ*, for hypothermic oxygenated perfusion (HOPE) [9, 10, 11, 12] and normothermic machine perfusion (NMP) [13, 14, 15, 16, 17, 18]. Compared with static cold storage (SCS), dynamic organ preservation reduces ischemia after or during procurement, allows for graft assessment, and can even enable repair and reconditioning of injured and diseased allografts [19]. Machine perfusion is now widely implemented for transplantation of kidneys, lungs, hearts, and livers following demonstration of non‐inferiority in clinical trials [14, 17, 20, 21]. Compared with SCS, HOPE prolonged total preservation time from 16 to 28 h in kidneys [22, 23], from 4 to 12 h in hearts [24, 25], and from 12 to 20 h in livers [26, 27], while NMP and successful transplantation were performed for livers after up to 68 h of perfusion [28], for lungs after more than 12 h of NMP [29], and for hearts after more than 9 h of NMP [30]. Importantly, the use of machine perfusion improved utilization of donation after circulatory death (DCD) and extended criteria donor grafts significantly [31, 32]. Safety of DCD transplantation was further improved by leveraging machine perfusion as a platform for real‐time graft assessment [33, 34, 35]. For livers, prolonged perfusion further promises absorption of ischemia–reperfusion injury with associated inflammatory molecules [36] and even repair of steatotic liver grafts, which is now shown in a clinical trial [37, 38]. Remarkably, NMP even allowed for transplantation of DCD hearts that were resuscitated *ex situ* [15].

Despite these advances in machine perfusion, the full demand for organs remains unmet. Therefore, the transplant community is exploring alternative organ sources. In this context, the transplantation of genetically modified organs from non‐human sources is one alternative for deceased donor donation and is recently explored in clinical settings [39, 40, 41, 42, 43]. The first genetically modified porcine kidneys and livers were transplanted into brain‐dead patients with promising results [44, 45]. This led to the later transplantation of a porcine liver as an auxiliary graft in a living recipient [43] and two transplants of porcine kidneys into living patients at New York University and Massachusetts General Hospital [41, 42]. Further, two pig‐to‐human heart transplants were successfully performed, but patient survival was limited to 60 and 40 days after transplantation [39, 40].

Despite the ability to procure high‐quality porcine organs from anesthetized living animals, *ex situ* perfusion becomes a critical tool for successful implementation of xenotransplantation in clinical practice. On one hand, perfusion enables mitigation of ischemia during organ transport, which is inherently longer than for human grafts, since there are only a few breeders of genetically modified pigs. On the other hand, *ex situ* perfusion becomes a critical tool for graft assessment, not only in terms of function but also with respect to zoonotic infections, which must be ruled out before transplantation. Lastly, we might see a renaissance of xenogenic cross‐circulation with porcine liver grafts as a strategy to bridge patients with liver failure to a transplant—a strategy that relies critically on appropriate machine perfusion systems. Therefore, we discuss the use of *ex situ* machine perfusion in the context of xenotransplantation in this article. Here, we define xenoperfusion as the perfusion of organs or tissues with blood or blood‐derived solutions obtained from another species than the organ donor. While xenoperfusion of liver grafts was recently discussed with a focus on preclinical research [46], we complement this article by highlighting the clinical impact of dynamic organ preservation as the field is now moving towards clinical implementation.

## 2 Machine Perfusion Modalities in Xenotransplantation

**FIGURE 1:**
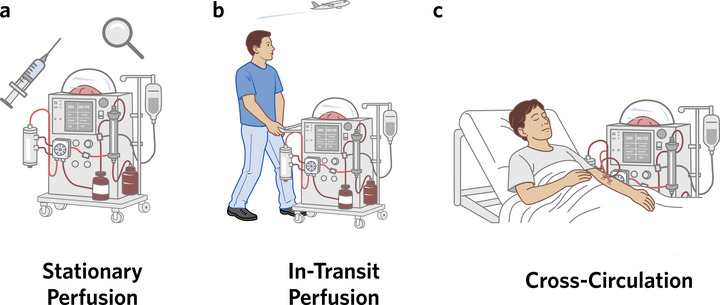
Xenoperfusion of genetically modified porcine grafts can be mostly applied in three main modalities. (a) Static use of machine perfusion at the transplant hospital to genetically modify wildtype grafts that fulfill transplant criteria, to assess graft function and viability, and to screen for zoonotic infections. (b) Dynamic preservation can also be used to minimize ischemia during transport from DPF breeders and procurement facilities to transplant centers. (c) In case of xenoperfusion of livers, perfusion systems can be connected to patients for temporary hepatic support to bridge patients to a transplant.

To date, perfusion technologies have been used primarily in clinical settings to minimize SCS [13, 17], extend preservation times, assess grafts [33], and potentially even repair and recondition marginal and extended‐criteria donor grafts [15, 47]. In the context of xenotransplantation, the use of perfusion technology offers alternative benefits. Given that procurement of porcine grafts can be scheduled based on demand, there is no need to bridge the night for a transplantation [48]. Furthermore, allografts can be procured from anesthetized living pigs, rendering high‐quality grafts with minimal ischemia and no underlying diseases. Therefore, there is less demand for *ex situ* perfusion to repair marginal and injured grafts. We identify three modalities that can benefit from machine perfusion in xenotransplantation (Figure 1).

First, *ex situ* perfusion can be applied in a stationary fashion, where a porcine graft is perfused at the site of transplantation. Here, multi‐day perfusion can be leveraged to genetically modify wildtype porcine organs from DPF breeders. Notably, long‐term NMP has facilitated successful AAV‐mediated gene therapy in donor livers [49, 50] and has further been employed to upregulate IL‐10 expression in human donor lungs through CRISPR‐Cas9 editing [51]. Although CRISPR‐mediated knockout of xenoantigens has not yet been demonstrated during *ex situ* perfusion, the mechanistic feasibility of such interventions has been established; suggesting that *ex situ* xenoantigen knockout is likely to be achieved in the near future. Long‐term perfusion is critical to this application, as it can take multiple days to knock out genes that are associated with graft rejection in xenotransplantation. Further, xenoperfusion can be used to screen for zoonotic infections and assess graft function prior to implantation.

Second, perfusion devices can be used to minimize ischemia after procurement, allowing for *ex situ* reoxygenation and longer transport without additional damage. This might be critical as there are only a few breeders of genetically modified pigs that are geographically dispersed. While NMP is favorable for stationary perfusion due to the required higher metabolic activity of the grafts, it remains unclear whether NMP or HOPE is more suitable for organ transport, as both have benefits. Routine implementation may further depend on cost, which is generally lower for HOPE, as NMP devices are more complex. Importantly, portable perfusion devices have to meet additional criteria to be transportable. As devices will not have access to hospital gas lines or power, they must incorporate battery packs with sufficient capacity. Further, gas supply for oxygen has to be provided during transport. Here, devices can either feature pre‐oxygenated perfusate that is solely circulated at hypothermic conditions (e.g., LifePort for Kidneys and Livers) [20, 52], use of a portable oxygen concentrator from ambient air (e.g., OrganOx) [17], or use of portable gas cylinders (e.g., Transmedics OCS) [18]. Transportable devices should be further built sufficiently compact to enable transport in cars, helicopters, and jets and should feature appropriate suspension to absorb shocks.

Beyond stationary use and portable use during transport, we further see an emerging use case for cross‐circulation, where a liver perfusion device is connected to a patient. Such extracorporeal liver perfusion may be a temporary life‐saving therapy for patients with acute liver failure and is discussed in a separate section as it is only applicable to livers.

## 3 Screening for Zoonotic Infections

Stationary use of machine perfusion creates a unique time window to rigorously assess xenografts for hours or even days prior to transplantation, not only with respect to graft function but also in terms of contaminations. Infectious safety is a defining translational gatekeeper for clinical xenotransplantation. Unlike allotransplantation, where donor‐derived infections largely involve known human pathogens, xenotransplantation introduces the possibility of cross‐species transmission of porcine microorganisms, including agents with uncertain or unknown pathogenicity in humans. Accordingly, contemporary frameworks emphasize a “defense‐in‐depth” strategy combining DPF breeding, longitudinal herd surveillance, donor‐specific screening at procurement, recipient monitoring, long‐term biobanking, and predefined infection‐control procedures for healthcare workers and close contacts [53, 54]. This systems‐level view is increasingly framed not as a single pre‐implantation checkpoint but as a continuous risk‐management process spanning source animal production to long‐term clinical follow‐up [53, 54]. Static donor testing has intrinsic limitations: it is time‐point dependent, may miss early incubation windows, can under‐detect latent/low‐level replication, and provides limited insight into whether a detected microorganism is biologically active under clinically relevant stressors such as reperfusion, warming, and exposure to human blood components. These limitations have motivated broader screening strategies that combine nucleic acid testing, serology, and functional assays and that explicitly recognize the need to detect not only the presence but also the behavior (activation and shedding) of relevant agents [53, 55].

Long‐term machine perfusion provides an ideal platform to conduct rigorous testing of procured organs, as it provides a multi‐day time window to monitor the graft and perform time‐intensive testing for infectious diseases. This further allows for monitoring of pathogen behavior, bridges potential initial incubation times, and enables detection of latent/low‐level pathogen replications. Thus, the utility of NMP goes beyond mitigation of ischemia but further improves immunological safety by adding an additional layer of infectious risk control prior to transplantation. Accordingly, xenoperfusion shifts infectious screening from a purely static donor‐based evaluation toward a dynamic biological assessment and intervention phase that precedes recipient exposure. Translating this dynamic screening concept into practice requires a precise definition of target pathogens and their corresponding diagnostic modalities. Primary infectious risks include endogenous retroelements such as porcine endogenous retroviruses (PERVs), latent DNA viruses prone to stress‐induced reactivation, including porcine cytomegalovirus/porcine roseolovirus (PCMV/PRV) and porcine lymphotropic herpesviruses, and adventitious pathogens introduced during organ procurement [39, 56, 57]. Effective *ex situ* screening mandates a multimodal diagnostic approach. This can involve rapid multiplex PCR for highly sensitive detection of established targets, unbiased metagenomic next‐generation sequencing (mNGS) to capture unexpected viral or bacterial sequences, and conventional microbial cultures to definitively exclude viable contamination [57]. Integrating these diagnostics fundamentally alters organ logistics. Crucially, perfusion duration must be intentionally prolonged, potentially to 24–48 h, to bridge the eclipse phase of viral replication. This extended window is essential to allow latent pathogens to reach detectable titers in the circulating perfusate, addressing a critical vulnerability identified in early clinical xenotransplants [58]. Consequently, capturing shedding kinetics requires standardized, longitudinal perfusate sampling at predefined intervals (e.g., baseline, 12, and 24 h). Finally, adopting these dynamic protocols necessitates updated regulatory frameworks that define acceptable limits of assay sensitivity, mandate specific multi‐pathogen screening panels, and establish real‐time decision algorithms for xenograft clearance prior to clinical implantation.

Most proposed screening programs prioritize viral risks, reflecting both experience in allotransplantation and the specific uncertainties of pig‐to‐human transfer. Guidance documents highlight porcine PCMV/PRV, porcine lymphotropic herpesviruses, hepatitis E virus (HEV), influenza A, and other regionally relevant swine viruses as high‐priority targets for DPF herd management and peri‐procurement testing [53, 54]. Endogenous retroelements, especially PERVs, remain a central regulatory concern because they are integrated in the pig genome and cannot be eliminated solely through biosecurity [59, 60]. While extensive clinical exposures and experimental work have not demonstrated easy transmission, the theoretical potential and the need for validated detection strategies continue to shape regulatory expectations and surveillance design [59, 60]. Previous studies have demonstrated the feasibility of PERV inactivation using CRISPR–Cas9–mediated genome editing in pigs, resulting in a substantial reduction of viral transmission potential [61]. The application of such genome‐editing strategies in the context of *ex situ* organ perfusion could therefore represent an additional layer of infectious risk control prior to transplantation.

## 4 Role of Xenoperfusion as a Research Platform

Beyond using *ex situ* perfusion for graft assessment and screening for infections, xenoperfusion provides a uniquely powerful research platform for xenotransplantation because it preserves intact organ physiology while allowing experimental control. Compared with in vivo models—where systemic confounders, ethical constraints, and limited sampling resolution often obscure mechanisms—machine perfused organs enable continuous, time‐resolved functional phenotyping under standardized boundary conditions. Traditional xenotransplantation research has relied predominantly on pig‐to‐nonhuman primate models, which, while indispensable, are constrained by systemic confounders, limited temporal resolution, and substantial ethical and logistical barriers. In contrast, *ex situ* NMP preserves intact vascular architecture, active metabolism, and organ‐specific function while allowing precise experimental control. This principle has been extensively validated in allotransplantation research, where perfused organs have become powerful platforms for functional assessment and therapeutic testing [17, 38, 62, 63, 64].

For xenotransplantation, this paradigm is particularly transformative, as early incompatibilities between porcine endothelium and human blood components often evolve within minutes to hours [65, 66]. Xenoperfusion enables continuous quantification of these early events through real‐time monitoring of vascular resistance, flow stability, oxygen consumption, metabolic activity, and organ‐specific functional outputs [67]. Machine perfusion provides an optimal platform for targeted *ex vivo* organ modification. Because perfusion preserves vascular access and metabolic activity, therapeutic agents can be delivered homogeneously throughout the organ at concentrations not achievable in vivo, followed by controlled washout prior to implantation [68]. This paradigm has been validated for pharmacological conditioning and increasingly for gene‐based interventions. Several studies have demonstrated the feasibility of viral vector delivery and functional gene expression during normothermic perfusion, establishing *ex situ* organ engineering as a realistic translational strategy rather than a theoretical concept [68, 69]. Xenoperfusion also broadens the framework for infectious safety assessment. By maintaining organs in a metabolically active state, perfusion allows the identification of pathogen activation, replication, or shedding processes that may remain undetected during SCS conditions. As understanding of many immunological processes involved in zoonotic disease transmission remains limited, NMP offers a unique research platform to study pathogen replication in the absence of the donor species' immune system. Serial sampling during especially long‐term perfusion enables longitudinal nucleic acid testing and facilitates the incorporation of metagenomic diagnostic approaches. This dynamic monitoring strategy directly addresses key concerns raised by infectious disease specialists regarding latent or stress‐induced zoonotic transmission [53, 54, 55].

Taken together, experimental findings indicate that xenoperfusion is more than just a method for organ preservation. Rather, it serves as a robust experimental platform for studying key aspects of xenotransplantation biology. It enables detailed analysis of endothelial–hematologic incompatibilities, functional comparison of genetically modified organs, and systematic evaluation of therapeutic strategies under controlled exposure to human blood. In this way, xenoperfusion fills an important translational gap between simplified in vitro systems and complex in vivo primate models, supporting mechanistic insight while limiting experimental variability and ethical burden.

## 5 Effect on Transport Logistics

So far, we have described the utility of stationary machine perfusion for research and rigorous assessment of grafts, which improves safety for xenotransplantation. Beyond stationary use, machine perfusion also constitutes a critical platform to mitigate ischemia during organ transport. A clinically scalable xenotransplantation ecosystem will depend not only on advances in genetic engineering and immunosuppressive strategies but equally on the establishment of a robust and reproducible logistical framework. In contrast to allotransplantation, where organs are procured from geographically distributed donor hospitals, xenotransplantation relies on a limited number of highly specialized DPF breeding facilities [70]. These facilities are subject to strict biosecurity regulations, continuous microbiological surveillance, and controlled housing conditions, which substantially restrict their geographic distribution. As a result, donor pigs are likely to be housed at centralized locations that may be separated by hundreds or even thousands of kilometers from recipient transplant centers. This spatial separation introduces a fundamental logistical challenge: xenogeneic organs must be transported over long distances under conditions that preserve graft viability, prevent risk of infection, and allow controlled integration into clinical workflows. Unlike allografts, where procurement and implantation are often performed under extreme time pressure, xenograft procurement can be scheduled electively given appropriate preservation strategies [48]. However, this advantage is offset by the necessity to ensure safe, traceable, and reproducible transport from farm to recipient.

In this context, xenoperfusion emerges not merely as a preservation technique but as a central enabling infrastructure for organ delivery [71]. Machine perfusion shifts organ transport from a passive, time‐limited process to an actively managed and information‐rich phase. Continuous perfusion allows maintenance of cellular metabolism, mitigation of ischemia–reperfusion injury, and real‐time monitoring of graft function during transit [33, 34, 35]. Parameters such as vascular resistance, flow dynamics, oxygen consumption, lactate clearance, acid–base balance, and metabolic trajectories can be continuously recorded, creating a longitudinal functional profile of the xenograft from procurement to implantation. This capability is particularly relevant in xenotransplantation, where early endothelial activation, coagulation dysregulation, and innate immune incompatibilities may develop rapidly and unpredictably [72].

Beyond preservation, xenoperfusion offers a unique opportunity to integrate functional screening into the transport phase. Exposure of the graft to human‐derived perfusates can serve as a pre‐implantation compatibility assay, revealing residual antibody binding, complement activation, or coagulation abnormalities prior to recipient implantation [73]. Such testing may substantially enhance safety by identifying grafts with unfavorable biological responses before irreversible clinical commitment. However, perfusate choice represents a critical determinant of transport strategy. Whole human blood perfusion provides the highest physiological relevance but is associated with substantial limitations, particularly in porcine liver grafts. Rapid sequestration and destruction of human erythrocytes, mediated by porcine Kupffer cells and lectin‐dependent recognition mechanisms, can lead to hemolysis, metabolic instability, and early graft dysfunction during prolonged perfusion [74]. In contrast, porcine blood‐based perfusion offers superior hemodynamic stability for long‐distance transport but provides limited insight into human‐specific immune compatibility [75]. Acellular and hemoglobin‐based perfusates reduce immunological interactions and logistical complexity, yet may insufficiently support long‐term metabolic function or endothelial homeostasis. These considerations suggest that xenotransplantation logistics may benefit from purpose‐driven, staged perfusion paradigms. For example, long‐haul transport from DPF farms could prioritize graft stabilization using porcine or optimized acellular perfusates, minimizing biological stress while ensuring metabolic viability. Near the recipient center, a controlled and time‐limited transition to human blood–based perfusion could then be implemented to assess functional compatibility under clinically relevant conditions. Such sequential perfusion strategies would balance logistical robustness with translational relevance.

Importantly, xenoperfusion also enables new organizational models for transplantation systems. Centralized organ production combined with standardized perfusion‐based transport could support huband‐spoke networks, in which multiple transplant centers receive organs from a limited number of certified breeding facilities [67]. Within such a framework, perfusion platforms become not only preservation devices but also standardized interfaces for quality control, data acquisition, and regulatory oversight. Harmonized datasets generated during transport could accelerate iterative refinement of genetic modifications, anticoagulation strategies, and immunosuppressive regimens. Collectively, these considerations position long‐term xenoperfusion as a cornerstone technology for clinical xenotransplantation. The success of the field will depend not solely on overcoming immunological barriers but on solving a systems‐level challenge in which organ logistics, perfusion technology, and biological compatibility are tightly interdependent. In this regard, xenotransplantation should be viewed not only as a problem of graft acceptance but as a problem of scalable organ delivery—one in which machine perfusion defines feasibility, safety, and clinical reach.

## 6 Xenogenic Liver Replacement Therapy—“Liver Dialysis”

Early extracorporeal approaches to liver failure were inspired by renal replacement therapy and focused primarily on toxin removal. These concepts evolved into albumin‐based dialysis systems, most prominently the Molecular Adsorbents Recirculating System (MARS) [76]. While MARS and related platforms improved biochemical parameters and hepatic encephalopathy, randomized trials failed to demonstrate consistent survival benefit, particularly in patients with acute‐on‐chronic liver failure (ACLF) [77, 78].

In parallel, bioartificial liver devices incorporating hepatocytes were developed to provide metabolic and synthetic support in addition to detoxification. The largest randomized clinical trial of a bioartificial liver in acute liver failure demonstrated feasibility but did not establish a definitive survival benefit, highlighting challenges related to cell mass, functional durability, and inflammatory host–device interactions [79].

A more physiologically ambitious approach emerged with extracorporeal perfusion of whole xenogenic livers, typically obtained from pigs, that is commonly referred to as “Xenogenic Liver Dialysis”. In a landmark clinical report from 1994, Chari and colleagues connected patients with fulminant hepatic failure to an *ex situ* perfused porcine liver, demonstrating temporary metabolic support and technical feasibility [80]. However, these early clinical experiences occurred before the advent of modern genetic engineering, contemporary immunological insight, and structured infectious surveillance and were therefore limited by profound coagulopathy, immune activation, and safety concerns [81]. Nevertheless, these studies established a principle that continues to guide current translational efforts: an intact organ can fully replicate the liver's systems‐level physiology, including dynamic metabolic adaptation and complex inter‐organ communication. Therefore, the concept of xenogenic cross‐circulation was further pursued.

Early preclinical studies demonstrated that isolated porcine livers could be maintained *ex situ* under normothermic conditions while preserving key metabolic functions, including ammonia clearance, urea synthesis, lactate metabolism, and bile production [82, 83]. In large‐animal models, extracorporeal porcine liver perfusion connected to recipient circulation resulted in measurable detoxification capacity and partial correction of metabolic derangements associated with acute liver failure [80, 84, 85, 86]. Several experimental systems have explored the concept of xenogenic liver dialysis, in which venovenous extracorporeal circuits routed recipient blood or plasma through a perfused porcine liver before reinfusion [87]. These models demonstrated rapid clearance of ammonia and bilirubin, improvement of acid–base balance, and transient stabilization of systemic hemodynamics. Importantly, these effects were achieved without permanent implantation of xenogenic tissue, supporting the conceptual attractiveness of a temporary extracorporeal approach. However, these studies also consistently identified coagulation dysregulation as the principal limiting factor. Exposure of human or primate blood to porcine hepatic endothelium further resulted in profound platelet activation, thrombocytopenia, and consumptive coagulopathy, frequently necessitating early termination of perfusion [88, 89]. These observations highlighted the unique vulnerability of the liver in xenogeneic settings, given its intrinsic role in coagulation factor synthesis, platelet clearance, and innate immune surveillance.

The renewed interest in xenogenic liver replacement therapy is not driven by a change in clinical need but by fundamental advances in biological feasibility through permanent gene editing [90]. The development of multigene‐edited porcine donors targeting key mechanisms of hyperacute rejection, complement activation, innate immune recognition, and coagulation dysregulation has transformed the interface between porcine organs and human circulation [91, 92]. Mechanistic studies demonstrated that incompatibilities within the porcine coagulation and complement systems—particularly involving tissue factor expression, thrombomodulin dysfunction, and dysregulated protein C signaling—played a central role in these phenomena [93, 94]. These findings provided a strong biological rationale for targeted genetic modification of donor pigs, including expression of human thromboregulatory proteins and deletion of key xenoantigens. Subsequent preclinical work using genetically modified porcine livers has shown substantial improvement in hemocompatibility during *ex situ* perfusion, with prolonged maintenance of hepatic metabolic activity, and reduced platelet consumption compared with wild‐type organs [46, 95]. In perfusion circuits that incorporate modern anticoagulation strategies and machine perfusion technology, genetically engineered porcine livers demonstrated stable glucose metabolism, ammonia clearance, and bile secretion for extended periods [46, 67, 96]. Collectively, these studies established that the historical limitations of xenogenic liver dialysis were not purely technical but fundamentally biological, and that many of these barriers are now at least partially addressable through contemporary genetic and perfusion‐based approaches. Notably, 10 and 69 gene knockout pigs have been bred successfully by now and even demonstrated improved performance in individual xenotransplantation cases [43, 97].

So far, we have seen that neither technical approaches such as MARS nor bioartificial livers could rescue patients with acute liver failure. We have, however, established the technical feasibility of xenogenic liver dialysis, and can use modern gene editing to modify porcine organs. Therefore, we see increasing potential for extracorporeal liver perfusion and cross‐circulation to bridge patients who suffer from acute liver failure for a few days until they can receive an orthotopic allotransplant. Compared to temporary transplantation of a xenogenic porcine liver, this procedure does not require a surgery, thereby sparing the patient and refraining from any hemodynamic challenges that are associated with a transplant. Unlike in transplant settings where most perfusions are conducted for less than 24 h, use of xenoperfusion as an auxiliary graft for bridging a patient to a transplant would benefit from prolonged perfusion of the porcine liver graft.

Until recently, *ex situ* perfusion of liver grafts was limited to a few hours and longer perfusions would have damaged the porcine liver graft, compromising the health of the connected patient and potentially triggering inflammatory responses. Given substantial progress in engineering of highly automated NMP devices, livers can now be perfused for up to two weeks [75, 98, 99]. The concept of multi‐day *ex situ* liver perfusion was first reported in 2020, where a human liver was transplanted after more than 3 days of perfusion [28]. Several machine features have to be adapted from conventional NMP devices to enable multi‐day perfusion of liver grafts while maintaining the function of the graft to stabilize the patient. Building on previously published engineering design considerations for NMP devices [19, 100], we highlight here several aspects of particular relevance to xenogeneic liver dialysis.

**FIGURE 2:**
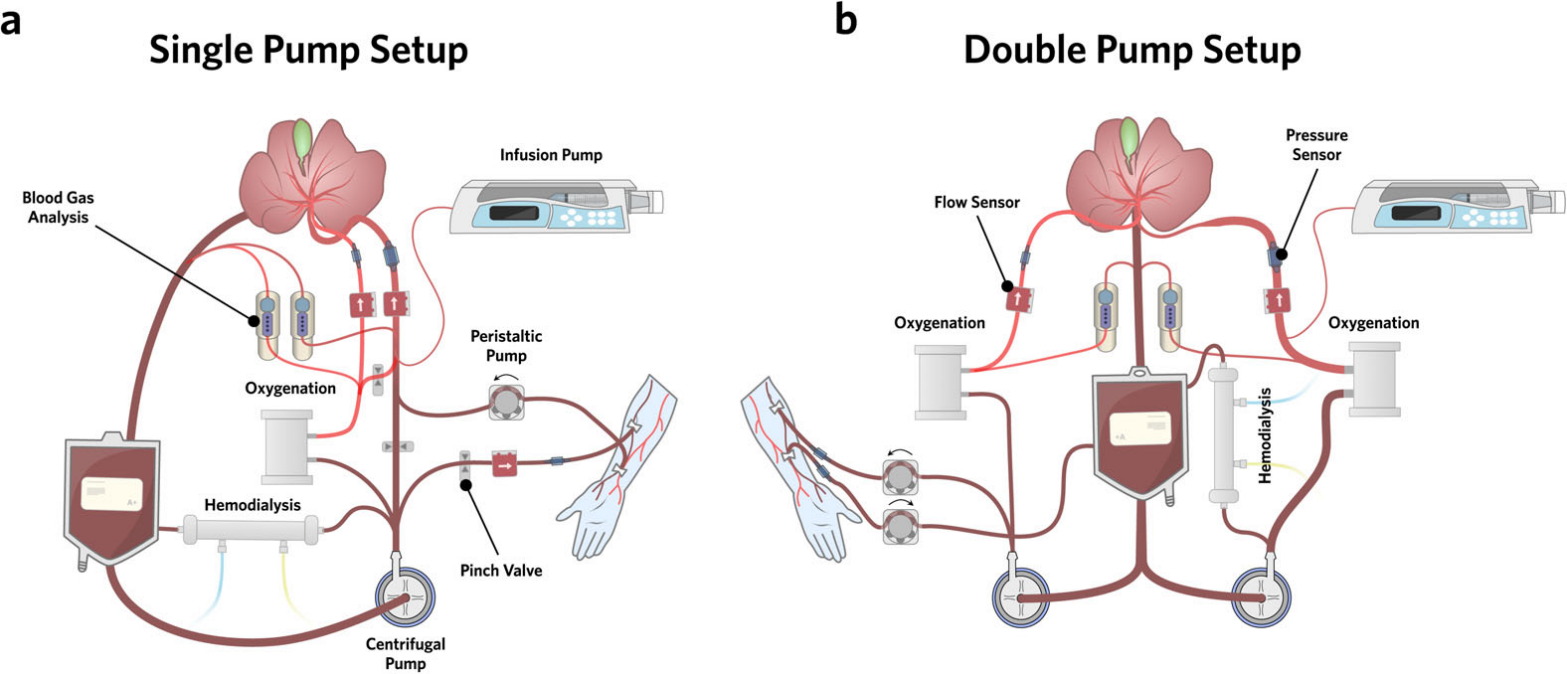
Perfusion setups for xenogenic liver dialysis that enable individual control of flow/pressure and oxygenation for the portal vein and the hepatic artery. (a) Single and (b) double pump setups for long‐term xenoperfusion of genetically modified porcine liver grafts to enable xenogenic liver dialysis. The major advantage of the single‐pump‐single‐oxygenator setup is reduced cost and lower hemolysis, while use of the double‐pump‐double‐oxygenator setup is more intuitive and requires less automated control. Vascular access is given via an AV fistula in the patient.

Given that the liver has a dual blood supply, perfusion devices need to account for different pressure and oxygenation in both afferent vessels. There are two main engineering approaches to modulate pressures in both the portal vein and hepatic artery. First, there is a single pump approach (Figure 2a), where portal venous flow is controlled with the perfusate pump, and the hepatic arterial pressure is regulated with an electrical pinch valve that pinches the tubing leading to the portal vein [75]. Secondly, there is an approach that uses two separate perfusate pumps, where flow and pressure is controlled by setting the respective pump speeds [101] (Figure 2a).

Importantly, hyperoxia should be avoided in the portal vein, as it causes vasoconstriction in the hepatic artery and impairs liver health during long‐term perfusion [102]. Lower oxygenation in the portal vein can be achieved by either using two oxygenators [101] or by using an electrical pinch valve to mix arterial blood with deoxygenated venous blood [75]. Another important design consideration for long‐term perfusion of porcine liver grafts is the choice of an appropriate perfusate pump. While peristaltic pumps are commonly used in setups for HOPE, centrifugal pumps have better performance for higher flow rates at normothermic conditions. The use of centrifugal pumps creates less shear forces on erythrocytes, thereby reducing hemolysis. In addition, centrifugal pumps allow for controlled pulsatile flow which further reduces hemolysis compared to constant flow rates [100]. Another important feature of long‐term perfusion devices is the incorporation of a hemodialysis loop [75, 102, 103]. Especially when establishing cross‐circulation with a patient, electrolyte balance should be provided to spare the kidneys of the patient. Vascular access to the patient can be implemented analogously to renal replacement therapy, for example by using an AV fistula [105]. Importantly, vessel pressure should be monitored in real‐time to avoid pressure increases in the veins of the patient. Further, flows should be controlled and monitored. This can be implemented by using peristaltic pumps at low flow rates, which directly control the flow, but expose erythrocytes to shear forces. Alternatively, flow into the patient's vein can also be controlled with an electrical pinch valve that sets a desired flow/pressure.

Taken together, the community has now established the technological requirements, from a biological and engineering point of view, to make xenogenic liver dialysis an attractive treatment option for patients with acute liver failure. Especially because this approach is only intended to bridge the patient for a few days, it might not be necessary to genetically modify the entirety of liver proteins to bring patient benefit. Most recently, biological and technological advances have culminated in the first human decedent study of xenogenic liver dialysis using multigene‐edited porcine livers, demonstrating sustained whole‐organ metabolic and synthetic support over several days with limited immunosuppression [106]. In contrast to conventional liver dialysis systems that are restricted to detoxification, this study provided proof of principle that xenogenic liver dialysis can transiently replace essential hepatic functions at a systems level, including synthesis of coagulation factors, thereby redefining the conceptual boundaries of xenogenic liver dialysis toward true temporary liver replacement. Shaked et al. demonstrated sustained hepatic function of xenografts for over 3 days in three human decedents with the native liver in situ, and for over 2 days in a decedent that underwent hepatectomy. Decedents were minimally immunosuppressed with methylprednisolone only, and inflammatory infiltration mainly consisted of macrophages and monocytes with only a few CD3+ T cells that infiltrated periportal regions [106].

## 7 Limitations and Remaining Challenges

While machine perfusion holds significant promise for the clinical translation of xenotransplantation, several challenges remain. First, machine perfusion is not yet standard practice in all transplant centers, and its implementation requires substantial resources, including specialized equipment, trained personnel, and dedicated infrastructure. The financial burden of establishing perfusion capabilities and performing prolonged perfusion—whether for infection screening, genetic modification, or xenogenic liver dialysis—may prevent smaller centers from participating in xenotransplantation programs. A major technical limitation is the absence of commercially available perfusion devices certified for use beyond 24 h. This temporal constraint restricts the feasibility of comprehensive infection screening, *ex situ* genetic modifications, and extended xenogenic liver dialysis. Conversely, currently certified devices are well‐suited for enhanced graft preservation during transport. In this context, the availability of such perfusion devices at procurement and DPF breeding facilities is essential. Notably, the optimal logistics for organ procurement and transport remain to be determined. Several organizational models are possible: breeders could establish dedicated procurement teams to manage transport to transplant centers; transplant centers could deploy their own procurement teams to breeding facilities; or specialized procurement organizations could coordinate both procurement and transport. The latter approach could leverage existing infrastructure, such as organ procurement organizations (OPOs) currently operating in the US, or involve the creation of xenograft‐specific organ procurement organizations (XOPOs). Regardless of the organizational model adopted, access to perfusion technology is critical for procurement teams, and financial constraints may limit the widespread adoption of machine perfusion in xenotransplantation.

## 8 Outlook

The rapid progression of xenotransplantation from prolonged preclinical primate studies to human applications marks one of the most dynamic translational advances in transplant biology in decades. Pioneering cases involving genetically modified porcine organs, most notably kidneys, have achieved unprecedented survival times in human recipients, with reports exceeding 270 days of function and substantial reduction in dependence on dialysis [107]. Regulatory bodies such as the U.S. Food and Drug Administration have now authorized clinical trials involving multiple pig kidney transplants in well‐characterized patient cohorts, signaling a transition from experimental case reports toward systematic evaluation [108]. In parallel, work in China has demonstrated extended kidney survival with genetically engineered grafts and has explored xenotransplantation beyond kidneys, including auxiliary liver implants that functioned for extended periods [45, 106]. These developments suggest that the next phase of xenotransplantation will be characterized by coordinated, multi‐organ clinical programs spanning multiple regulatory jurisdictions.

Machine perfusion is central to this evolution. *Ex situ* xenoperfusion not only preserves grafts but also creates a unique time window for observing organ physiology and immunology prior to recipient exposure. This capability could help define data‐driven transplant criteria, where viability parameters can be assessed following established frameworks [19]. Integration of real‐time sensors for measurement of surrogate biomarkers may permit early identification of incompatibilities that are invisible through static assays alone [34]. Importantly, xenotransplantation requires additional immunological assessment to clear a graft for safe transplantation. This includes cross‐species reactions as well as pathogens from the donor.

Beyond assessment, perfusion platforms open the possibility for functional organ conditioning and targeted *ex situ* modulation. Advanced delivery of gene therapy vectors, immunomodulatory small molecules, or localized complement and coagulation inhibitors could tailor grafts for individual recipients during NMP, based on immunological risk profiles. Such conditioning may reduce the necessary intensity of systemic immunosuppression post‐implantation and attenuate chronic rejection processes and the associated risks of infection.

Further, *ex situ* perfusion will be indispensable to mitigate ischemia during graft transport from DPF farms to local transplant centers. The flexibility, that is associated with preserving a graft on a perfusion device, further facilitates transplant logistics and improves coordination.

Given the metabolic complexity of the liver, including its synthetic function, it might still take a while to establish long‐term survival and function of transplanted porcine liver grafts in humans. However, given the encouraging results of recent auxiliary liver transplants [43, 45, 106], we might see clinical cases of xenogenic cross‐circulation soon. The main advantage of this approach is the minimal invasiveness due to simple vascular access, which spares the patient from additional injury. Further, species‐specific differences in liver proteins might not compromise long‐term outcomes, as this approach would be only used to bridge a patient for a few days until the liver either recovers, or the patient receives a human organ.

We conclude that machine perfusion systems allowing *ex situ* perfusion for several days, supported by real‐time analytics, multi‐omics data, and adaptive conditioning protocols, are poised to evolve from a preservation method into a central component of clinical xenotransplantation. Its integration into global clinical pathways will be essential to not only unlock the potential of xenografts in addressing organ shortage but also to ensure safety, reproducibility, and equitable access on a larger scale.

## Funding

Authors were supported by ETH Zurich, the FZ‐Healthcare Foundation, and the Liver and Gastrointestinal Disease (LGID) Foundation.
